# Salivary Oxidant/Antioxidant Status in Chronic Temporomandibular Disorders Is Dependent on Source and Intensity of Pain – A Pilot Study

**DOI:** 10.3389/fphys.2018.01405

**Published:** 2018-10-17

**Authors:** Ema Vrbanović, Iva Z. Alajbeg, Lea Vuletić, Ivana Lapić, Dunja Rogić, Ana Andabak Rogulj, Davor Illeš, Dubravka Knezović Zlatarić, Tomislav Badel, Ivan Alajbeg

**Affiliations:** ^1^Department of Removable Prosthodontics, School of Dental Medicine, University of Zagreb, Zagreb, Croatia; ^2^Department of Physiology, School of Dental Medicine, University of Zagreb, Zagreb, Croatia; ^3^Department of Laboratory Diagnostics, University Hospital Center Zagreb, Zagreb, Croatia; ^4^Department of Oral Medicine, School of Dental Medicine, University of Zagreb, Zagreb, Croatia

**Keywords:** orofacial pain, temporomandibular disorders, salivary diagnostics, oxidative stress, antioxidants, cortisol

## Abstract

Temporomandibular disorders (TMD) have been associated with altered salivary oxidative status, but the relation with pain source and pain severity isn’t clarified. With the aim to assess their interaction with TMD, we compared levels of selected salivary oxidative stress (OS) markers (glutathione peroxidase, superoxide dismutase, total antioxidant capacity (TAC), uric acid, 8-hydroxydeoxyguanosine, malondialdehyde) and salivary cortisol (SC) as a stress indicator, between 20 TMD patients and 15 healthy control subjects. In order to record differences relating to pain source and severity, patients were respectively classified according to specific diagnoses (myofascial pain or disc displacement (DD)), and pain intensity (high or low). TAC was significantly higher in TMD patients than in controls (morning *p* = 0.015; afternoon *p* = 0.005). Significant differences were also observed when TAC levels between high-intensity pain patients and controls were compared, as well as between DD patients and controls. In logistic regression analysis, higher levels of TAC were related to DD (morning OR: 1.66, 95%CI: 1.05–2.64, *p* = 0.029; afternoon OR: 2.10, 95%CI: 1.11–3.98, *p* = 0.021) and to high-intensity pain (morning OR: 1.81, 95%CI: 1.04–3.15, *p* = 0.037; afternoon OR: 1.79, 95%CI: 1.02–3.14, *p* = 0.043). We also found that morning SC was positively correlated with antioxidant parameters in TMD patients. Our data suggest compensatory mechanism as response to higher level of stress. This stress could be extrinsic and lead toward TMD, or intrinsic, emerging from established TMD, or could be both. The intensity and the source of pain should be considered important factors in future investigations evaluating salivary OS markers in TMD patients.

## Introduction

Oxidative stress (OS) has been implicated in the pathophysiology of many diseases, including oral diseases (Agha-Hosseini et al., 2012; Lopez-Jornet et al., 2014; Almerich-Silla et al., 2015; Babaee et al., 2016; Ahmadi-Motamayel et al., 2017; Kumar et al., 2017).

Different biospecimen, including saliva (Nagler et al., 2002; Chiappin et al., 2007), are used to assess the (dis)balance in oxidative status for its potential role in the onset and/or the progression of a disease. The role of OS has also been studied in temporomandibular disorders (TMD), the most common orofacial pain disorders of non-dental origin. Their etiopathophysiology is multifactorial, involving a combination of factors such as parafunctions, micro- and macro traumas, genetic influences, physiological and psychological stressors (Sharma et al., 2011). TMD mostly affect women between 20 and 40 years (Warren and Fried, 2001).

Because pain-related TMD impact individual’s daily activities, psychosocial functioning, and quality of life, it is important to understand their pathophysiology and the mechanisms involved in provoking pain and determining its intensity in order to determine factors predictive for disease severity and enhance therapeutic strategies for these patients. Advances in our understanding of the disorder and the mechanisms of pain allow the possibility of providing personalized care for TMD patients (Harper et al., 2016).

Several studies suggest that OS plays a role in the TMD and the experience of pain related to TMD. Rodríguez de Sotillo et al. (2011) reported increased OS products in TMD patients and a significant association between TMD pain and salivary OS markers. De Almeida and Amenábar (2016) determined lower total antioxidant capacity (TAC) in patients with pain-related TMD, but they found no correlation between TAC and pain intensity. Basi et al. (2012) as well as Etöz et al. (2012) support the role of OS in the intensity of pain in TMD but specimen other than saliva were used in these studies.

The aim of our study was to compare the levels of selected salivary OS markers and salivary cortisol (SC) between patients with chronic pain-related TMD and healthy controls and to assess differences relating to the source (muscle or joint) and the intensity (low or high) of pain. We also evaluated the relationship between OS markers and SC, a biological marker for stress response, since it is suggested that TMD patients have biological predisposition for enhanced stress reactivity (Rollman and Gillespie, 2000). On the other hand, TMD might impact cortisol levels (Jones et al., 1997), and cortisol changes might lead to altered redox changes (Aschbacher et al., 2013).

We hypothesized that we would encounter higher salivary oxidant levels and lower salivary antioxidant levels in TMD patients than in control group, depending on the source and intensity of pain, and that salivary OS markers would correlate with SC concentrations.

## Materials and Methods

### Subjects

This case-control study was performed at the School of Dental Medicine, University of Zagreb. The study was approved by the Ethics Committee (01-PA-26-6/15, item 3.2) and conducted in accordance with the Helsinki Declaration. All subjects were informed of the procedures involved in the study and provided written consent. Recruitment of participants was performed between May 2017 and April 2018.

Power analysis, performed to estimate sample size, was based on the data from the pilot study (Rodríguez de Sotillo et al., 2011). Minimal number of 30 participants (10 per group) was calculated with power set at 80% and a significance level of 5%. The effect size was hypothesized to be 0.58 based on previous studies.

Participants were recruited from patients seeking treatment for TMD and orofacial pain. Inclusion criteria were painful disc displacement (DD) or myofascial pain (MP) according to the diagnostic criteria for TMD (DC/TMD) (Schiffman et al., 2014) and reports of ongoing pain lasting more than 6 months.

Exclusion criteria were other orofacial pain conditions including dental pain, smoking, poor oral hygiene, gum swelling, periodontitis, oral lesions, chronic medical conditions (cardiovascular disease, cancer, diabetes, autoimmune diseases), pregnancy, use of supplements and medications known to affect the results of our tests. Patients displaying combined MP and DD and patients with degenerative joint disease were also excluded. Twenty TMD patients and 15 healthy age matched control subjects were included. All participants were women. Different groups relating to pain source (10 DD and 10 MP) were formed in order to explore its interaction with OS.

### Assessment of Pain Intensity

Characteristic pain intensity (CPI) was assessed using the graded chronic pain scale by computing the means of three items (current pain, worst pain, average pain) and multiplying them by 10. CPI < 50 was considered to be low-intensity pain (LIP), and CPI ≥ 50 was considered to be high-intensity pain (HIP). Subsequently, we formed another division of TMD patients according to pain severity (10 HIP and 10 LIP).

### Sample Collection

The methods of sample collection and analysis were described in detail and validated by our group in a prior study (Alajbeg et al., 2017). The subjects were instructed to fast before saliva collection in the morning and to not eat or drink anything but water at least 2 h before sampling in the afternoon. Brushing teeth before saliva sampling was forbidden to avoid blood contamination. Five mL of whole, unstimulated saliva sample was collected in a graduated tube (50 mL, self-standing centrifuge tubes, Ratiolab, Germany). Saliva aliquots (1 mL) were stored at −80°C until analysis. Since some of the markers showed significant diurnal variations, saliva was collected both in the morning (7 AM) and in the afternoon (5 PM).

### Salivary Analysis

Saliva samples were used to analyze the following OS markers: 8-hydroxydeoxyguanosine (8-OHdG), malondialdehyde (MDA), TAC, glutathione peroxidase (GPX), superoxide dismutase (SOD), and uric acid (UA). For these assays, analytical performance, including intra- and inter-assay variability, was assessed. The data and a detailed description of the methodology is available in our previous study (Alajbeg et al., 2017).

Free SC was analyzed using a competitive ELISA kit (Demeditec Diagnostics GmbH, Germany). The intra- and inter-assay variabilities of this assay kit are 5.8 and 6.4%, respectively, according to the manufacturer. All results were normalized to the total protein concentration. Sample analysis was performed at the Department of Laboratory Diagnostics, University Hospital Center Zagreb.

### Statistical Analysis

Analyses were performed using SPSS 17.0 (Chicago, IL, United States) with the alpha set at *p* < 0.05. Data distribution was tested using the Shapiro-Wilk test. Student’s *t*-test was used for comparison of two groups (TMD vs. controls) and analysis of variance (ANOVA) for comparison of three groups (controls vs. TMD subgroups), for the normally distributed data (age and CPI). Mann-Whitney U test and Kruskal-Wallis test were used for comparison of two and three groups, respectively, if the data were not normally distributed (OS markers and SC).

Spearman correlation evaluated the association between salivary OS products and antioxidative enzymes with SC. Logistic regression analysis determined the association between each marker, which were the independent variables, and the groups, for which the dependent variables were study groups (TMD and controls), source of pain (MP or DD), and pain intensity (LIP or HIP).

## Results

No statistically significant age differences were noted between 20 TMD subjects (39.30 ± 12.07) and 15 controls (34.33 ± 7.86) (t = −1.38; *p* = 0.175) nor between the control subjects and the TMD subgroups of 10 MP (42.6 ± 12.65) and 10 DD (36.00 ± 11.10) subjects (F = 2.01, *p* = 0.15). The mean CPI of MP subjects (46.03 ± 15.92) and DD subjects (45.4 ± 19.77) were not statistically different (t = 0.078, *p* = 0.938). Five MP and 5 DD subjects had LIP (34.2 and 28.2, respectively). The difference between groups was non-significant (t = 0.963, p = 0.36). Similarly, no significant difference (t = −0.819, *p* = 0.43) was shown between 5 MP and 5 DD subjects with HIP (57.8 and 62.6, respectively). Therefore, patients were pooled in pain intensity subgroups regardless of the source. Conversely, patients were pooled in pain source subgroups regardless of pain intensity.

The levels of salivary GPX among TMD subjects were significantly lower than among the controls (morning: Z = 2.43; *p* = 0.014; afternoon: Z = 1.98; *p* = 0.047). The levels of salivary TAC were significantly higher in TMD patients compared to the controls (morning: Z = −2.41; *p* = 0.015; afternoon: Z = −2.76; *p* = 0.005). Morning SC was significantly higher (Z = −2.01, *p* = 0.043) in the TMD group than in the control group. Differences in salivary OS markers and SC between the control group and TMD subgroups are presented in **Table 1**.

**Table 1:** Differences in salivary oxidative stress markers and salivary cortisol between the control group and TMD subgroups.

	Marker	Control (*N* = 15)		TMD (*N* = 20)							
				Diagnosis (source)				Pain intensity			
				MP (*N* = 10^∗^)		DD (*N* = 10^∗^)		HIP (*N* = 10^∗∗^)		LIP (*N* = 10^∗∗^)	
		Mean	(95%CI)	Mean	(95%CI)	Mean	(95%CI)	Mean	(95%CI)	Mean	(95%CI)
**Morning**	GPX (U/g)	91.74^AC^	(56.05–126.88)	71.57	(17.77–125.38)	41.61^A^	(12.08–71.15)	66.23	(18.83–113.63)	46.96^C^	(6.08–87.82)
	SOD (U/g)	2873.48	(2167.5–3579.4)	3196.38	(1227.7–5165.1)	5773.14	(2327.21–9219.1)	6255.86	(2761.7–9749.9)	2713.66	(1349.1–4078.2)
	TAC (mmol/g)	2.65^AD^	(1.743–3.56)	4.94	(1.9–7.98)	6.01^A^	(3.2–8.82)	6.93^D^	(3.55–10.3)	3.82	(242–5.23)
	UA (μmol/g)	375.12	(223.39–526.84)	670.54	(137.16–1203.92)	619.43	(337.67–901.20)	825.94	(278.38–1373.49)	464.04	(297.96–630.12)
	8-OHdG (μg/g)	1.94	(0.98–2.91)	1.24	(0.3–2.21)	2.29	(0.52–4.06)	2.31	(0.45–4.17)	1.22	(0.46–1.99)
	MDA (nmol/g)	329.97	(115.40–544.54)	341.65	(58.79–624.51)	483.30	(23.41–990.04)	635.37	(123.03–1147.71)	189.59	(36.31–342.87)
	SC (μg/g)	24.16	(15.11–33.21)	43.74	(22.19–65.28)	38.40	(18.91–57.89)	49.59	(24.92–74.25)	32.55	(19.85–45.26)
**Afternoon**	GPX (U/g)	141.35	(35.59–247.12)	52.16	(29.21–75.10)	61.50	(32.72–90.28)	50.95	(27.42–74.46)	62.72	(34.67–90.77)
	SOD (U/g)	2646.70	(1603.6–3689.7)	2424.7	(631.73–4217.7)	3797.8	(2189.8–5405.7)	3574.17	(1832.9–5315.4)	2648.35	(898.9–4397.8)
	TAC (mmol/g)	2.93^ACD^	(1.83–4.03)	4.65^B^	(2.49–6.81)	6.39^AB^	(3.79–8.98)	5.49^D^	(3.35–7.64)	5.45^C^	(2.65–8.25)
	UA (μmol/g)	402.32	(284.12–520.53)	638.20	(197.33–1079.08)	795.18	(302.81–1287.56)	722.55	(304.16–1140.93)	710.84	(192.45–1229.23)
	8-OHdG (μg/g)	2.06	(1.03–3.08)	1.37	(0.2–2.56)	2.50	(0.82–4.18)	2.18	(0.77–3.59)	1.69	(0.2–3.29)
	MDA (nmol/g)	727.55	(359.38–1095.71)	738.29	(136.57–1613.16)	1209.99	(704.39–1715.61)	1090.55	(201.79–1979.31)	857.74	(329.54–1385.93)
	SC (μg/g)	7.16	(3.34–10.98)	6.55	(2.27–10.83)	14.11	(2.91–25.32)	9.15	(3.32–14.98)	11.51	(0.34–22.68)

*^∗^Pooled regardless of pain intensity; ^∗∗^pooled regardless of pathology source (diagnostic subgroup). TMD, temporomandibular disorders; MP, myofascial pain; DD, disc displacement; HIP, high-intensity pain; LIP, low-intensity pain; GPX, glutathione peroxidase; SOD, superoxide dismutase; TAC, total antioxidant capacity; UA, uric acid; 8-OHdG, 8-hydroxydeoxyguanosine; MDA, malondialdehyde; SC, salivary cortisol; CI, Confidence Interval; Mann-Whitney U test was used for comparisons. ^A^P-values between control vs. DD: P_morning GPX_ = 0.012, P_morning TAC_ = 0.027, P_afternoon TAC_ = 0.005. ^B^P-values between MP vs. DD: P_afternoon TAC_ = 0.041. ^C^P-values between control vs. low-intensity pain: P_morning GPX_ = 0.011, P_afternoon TAC_ = 0.027. ^D^P-values between control vs. high-intensity pain: P_morning TAC_ = 0.012, P_afternoon TAC_ = 0.016.*

In TMD patients, antioxidant parameters were positively correlated with morning SC (**Table 2**). Among control subjects, no significant correlation was found between SC and OS markers.

**Table 2:** Correlations^∗^ between salivary cortisol and salivary oxidative stress markers.

				Marker					
				GPX (U/g)	SOD (U/g)	TAC (mmol/g)	UA (μmol/g)	8-OHdG (μg/g)	MDA (nmol/g)
Control (*N* = 15)	Morning	SC (μg/g)		−0.22	0.23	0.01	−0.02	0.24	−0.33
	Afternoon			−0.22	0.08	0.40	0.30	0.11	0.05
TMD (*N* = 20)	Morning			0.59^A^	0.45^B^	0.64^C^	0.45^D^	0.19	0.39
	Afternoon			−0.07	0.35	0.39	0.32	0.20	0.08

*^∗^Spearman correlation coefficient; GPX, glutathione peroxidase; SOD, superoxide dismutase; TAC, total antioxidant capacity; UA, uric acid; 8-OHdG, 8-hydroxydeoxyguanosine; MDA, malondialdehyde; SC, salivary cortisol. ^A^P-value = 0.005. ^B^P-value = 0.044. ^C^P-value = 0.002. ^D^P-value = 0.044.*

TAC was positively associated with TMD (morning OR: 1.66, 95%CI: 1.03–2.66, *p* = 0.034; afternoon OR: 1.86, 95%CI: 1.07–3.21, *p* = 0.025).

When evaluating the association between each salivary OS marker and the source of pain, higher levels of TAC in the morning (OR: 1.66, 95%CI: 1.05–2.64, *p* = 0.029) and in the afternoon (OR: 2.10, 95%CI: 1.11–3.98, *p* = 0.021) were positively associated with DD. No such association was found for MP. Higher TAC was also related with HIP (morning OR: 1.81, 95%CI: 1.04–3.15, *p* = 0.037; afternoon OR: 1.79, 95%CI: 1.02–3.14, *p* = 0.043).

## Discussion

The results of our study did not confirm lower levels of salivary TAC in TMD patients as we expected, based on the results of previous studies (Rodríguez de Sotillo et al., 2011; De Almeida and Amenábar, 2016). On the contrary, TAC was significantly higher in our TMD patients in comparison with controls. Considering that they experienced pain lasting more than 6 months, higher TAC might imply a compensatory increase of the antioxidant enzymes in response to changing levels of OS as a prerequisite for efficient defense (Sies, 1993). De Almeida and Amenábar (2016) did not report how long their patients experienced TMD pain, and it is unclear from the report by Rodríguez de Sotillo et al. (2011) whether alltheir patients had chronic pain. With longer duration of the disorder, antioxidant systems might regenerate (if their level was decreased in comparison with healthy subjects) or increase, probably as a compensatory mechanism (as found in our study). Duration of the TMD might thus, at least partly, explain why this result differs from previous studies. TAC increase could be greater in patients experiencing more severe symptoms, as suggested by our finding of higher TAC levels in subjects with HIP. However, individual antioxidants might show less ability to adapt, as suggested by the finding of a significantly lower GPX in TMD patients. Regarding the source of pain, higher TAC was positively related only to pain of joint origin.

Rodríguez de Sotillo et al. (2011) reported significantly higher levels of 8-OHdG and MDA in TMD patients, and an association between higher levels of these OS markers with higher scores of pain intensity. In our study, the concentrations of MDA and 8-OHdG were higher in specific subgroups of TMD patients (HIP and DD) compared to controls, but the difference was not statistically significant. Previous investigations did not report salivary biomarker levels normalized for the total protein concentration in saliva, which partly explains the inconsistencies between our results and those of previous studies.

The mechanisms by which OS may modulate pain in TMD are not known but are probably diverse. Ray et al. (2015) showed that the production of oxidatively modified lipoproteins induces nociception and Medow et al. (2013) suggested direct alteration of local sensory nerve activity by certain reactive oxygen species as a mechanism of their influence on the generation and maintenance of pain-associated symptoms and myalgias in subjects with chronic fatigue syndrome. The latter finding is particularly interesting in the light of a strong clinical association between muscular TMD and fibromyalgia and chronic fatigue syndrome as reported by Korszun et al. (1998). This association relates to the perturbation of the hypothalamic-pituitary-adrenal axis and subsequent hyperreactivity to stress which then manifests as one or more stress-related conditions. Higher SC levels and a positive correlation of antioxidants with SC in TMD patients, observed in our study, might indicate that stress either has a role in the development of TMD, or that pain-related TMD might produce additional stress to the body and further enhance cortisol secretion.

Number of subjects and high variability of OS markers clearly represent study’s limitations, and demand caution in the interpretation of results. Nevertheless, results encourage research on the relationship between OS and TMD, aiming to better understand the disturbed antioxidative/oxidative balance and TMD, either as the consequence or the cause of one another. In conclusion, both the intensity and source of pain as well as time of saliva sampling should be considered in future investigations to determine the diagnostic utility of OS markers in the saliva of TMD patients.

## Data Availability

The datasets generated and analyzed during the current study are not publicly available, because this is a pilot study and further study will include more TMD patients who are still being recruited. However, all data from this study is available from the corresponding author on reasonable request.

## Author Contributions

EV and LV collected the samples and wrote the manuscript. IZA designed the study, analyzed the data, and wrote the manuscript. IL measured the samples and co-wrote the manuscript. DR contributed substantially to the concept and design, and measured the samples. AAR collected the samples and coordinated specimen storage. DI analyzed and interpreted the data. DKZ collected the samples and analyzed the data. TB planned the analyses and collected the samples. IA designed the study and critically reviewed the manuscript.

## Conflict of Interest Statement

The authors declare that the research was conducted in the absence of any commercial or financial relationships that could be construed as a potential conflict of interest.
